# TransformerGO: predicting protein–protein interactions by modelling the attention between sets of gene ontology terms

**DOI:** 10.1093/bioinformatics/btac104

**Published:** 2022-02-17

**Authors:** Ioan Ieremie, Rob M Ewing, Mahesan Niranjan

**Affiliations:** Vision, Learning & Control Group, University of Southampton, Southampton SO17 1BJ, UK; Biological Sciences, University of Southampton, Southampton SO17 1BJ, UK; Vision, Learning & Control Group, University of Southampton, Southampton SO17 1BJ, UK

## Abstract

**Motivation:**

Protein–protein interactions (PPIs) play a key role in diverse biological processes but only a small subset of the interactions has been experimentally identified. Additionally, high-throughput experimental techniques that detect PPIs are known to suffer various limitations, such as exaggerated false positives and negatives rates. The semantic similarity derived from the Gene Ontology (GO) annotation is regarded as one of the most powerful indicators for protein interactions. However, while computational approaches for prediction of PPIs have gained popularity in recent years, most methods fail to capture the specificity of GO terms.

**Results:**

We propose TransformerGO, a model that is capable of capturing the semantic similarity between GO sets dynamically using an attention mechanism. We generate dense graph embeddings for GO terms using an algorithmic framework for learning continuous representations of nodes in networks called node2vec. TransformerGO learns deep semantic relations between annotated terms and can distinguish between negative and positive interactions with high accuracy. TransformerGO outperforms classic semantic similarity measures on gold standard PPI datasets and state-of-the-art machine-learning-based approaches on large datasets from *Saccharomyces cerevisiae* and *Homo sapiens*. We show how the neural attention mechanism embedded in the transformer architecture detects relevant functional terms when predicting interactions.

**Availability and implementation:**

https://github.com/Ieremie/TransformerGO.

**Supplementary information:**

Supplementary data are available at *Bioinformatics* online.

## 1 Introduction

Identifying protein–protein interactions (PPIs) is a major challenge in molecular biology, because it is fundamental for our understanding of biological processes (BPs) and cellular activities, such as metabolism, signal transduction pathways and immune response. Advances in high-throughput methods allowed the discovery of PPIs at the genome scale. However, experimental methods are time-consuming, labour-intensive and the results suffer from high false positive and negative rates. Yeast-two-hybrid experiments (Ito *et al.*, 2001) report direct physical interactions and generate binary interactome network maps. The noise found in the final datasets comes primarily from the inability of the method to capture interactions between proteins that rely on intermediary proteins (protein complexes and post-translational modifications) and on expression levels. On the other hand, experiments using affinity-purification and mass spectrometry (AP-MS) (Ewing *et al.*, 2007; Gavin *et al.*, 2006) generate datasets complementary to the previously mentioned method by detecting interactions appearing in protein complexes. However, AP-MS is limited in its ability to detect transient interactions (Cafarelli *et al.*, 2017). Therefore, computational approaches have been developed to infer PPIs *in silico*.

Multiple studies approached the prediction of PPIs using various sources of information, such as the primary structure of the protein (Chen *et al.*, 2019; Hashemifar *et al.*, 2018; Li *et al.*, 2018), the 3D protein structure (Bepler and Berger, 2021), gene expression profiles (Chin *et al.*, 2010) and Gene Ontology (GO) annotation (Bandyopadhyay and Mallick, 2017; Jain and Bader, 2010; Kulmanov *et al.*, 2019; Smaili *et al.*, 2018, 2019; Zhang *et al.*, 2018, 2020).

The GO project is a collaborative effort to annotate genes and the products of genes with useful descriptions of BPs across multiple databases and species (Gene Ontology Consortium, 2015). GO is composed of the ontology graph and annotation databases. The graph is structured as a directed acyclic graph (DAG) and is divided into three orthogonal sub-ontologies, cellular component (CC), BP and molecular function (MF). Nodes inside the graph denote GO terms, which are descriptions of biological concepts and the edges (‘is_a’, ‘part_of’, ‘regulates’, ‘has_part’) represent relations between GO terms (Gene Ontology Consortium, 2015). Annotation databases contain GO terms and the gene products they annotate to. The semantic similarity in GO annotation is regarded as one of the most powerful descriptors of PPIs (Gene Ontology Consortium, 2015; Miller *et al.*, 2005; Patil and Nakamura, 2005). The idea behind this is that interacting protein pairs, such as protein complexes interact in the same cellular location and functional modules participate in the same cellular processes or MFs at different times. These two types of interactions are closely related in terms of GO annotation (Zhao *et al.*, 2020).

Multiple semantic similarity measures on GO have been proposed over the years (Jain and Bader, 2010; Resnik, 1995; Zhang *et al.*, 2018) that predict PPIs using semantic similarity in GO annotation. However, classic semantic similarity measures are in general handcrafted and fail to fully capture the specificity of GO terms. It has also been shown that semantic similarity measures are difficult to compare and are only performing well on some datasets (Kulmanov and Hoehndorf, 2017). Depending on the downstream application, different features should be more or less relevant in defining the notion of similarity (Smaili *et al.*, 2018). On the other hand, while machine-learning approaches (Jansen *et al.*, 2003; Rhodes *et al.*, 2005; Stelzl *et al.*, 2005) can be trained in a supervised fashion, the similarity is encoded as a simple feature vector indicating the common GO terms. Disregarding the structure of the ontology would not allow for a correct evaluation of proteins that have common terms but are too general (Guo *et al.*, 2006). Several studies apply techniques from the field of Natural Language Processing to extract dense feature vectors for GO terms (Smaili *et al.*, 2018, 2019; Zhang *et al.*, 2020; Zhao *et al.*, 2020; Zhong *et al.*, 2019). We find that previous work comparing feature vectors using cosine similarity or using a fully connected neural network fail to capture deep semantic similarity between the GO terms.

Inspired by previous work based on GO terms and current advancements made in NLP, we propose a trainable approach called TransformerGO that predicts PPIs using information extracted from the GO graph. We apply node2vec (Grover and Leskovec, 2016) to generate dense feature vectors for GO terms and then use the Transformer model (Vaswani *et al.*, 2017) to dynamically learn a deep semantic similarity between sets of GO terms. We demonstrate that TransformerGO outperforms classic similarity measures and recent models that use a similar way of encoding the GO graph. Furthermore, experiments that analyse the attention weights show how semantic similarity is learned in the decoder and provide useful visualizations that could aid future research on comparing proteins at a functional level.

## 2 System and methods

### 2.1 Protein embeddings

With recent research on unsupervised representation learning, new methods for creating latent representations of nodes and edges in networks have emerged. DeepWalk (Perozzi *et al.*, 2014) uses information from truncated random walks to learn link features by extending the Skip-gram model (Mikolov *et al.*, 2013). The network is represented as a document, and the nodes in the random walks are the equivalent of words forming sentences. Node2vec (Grover and Leskovec, 2016) is a framework that learns continuous feature representations for nodes by maximizing the log-likelihood of preserving the network neighbourhoods. Compared to the previous method, Node2vec adds flexibility when defining the neighbourhoods by using biased random walks. The model has proven successful in multi-label classification and link prediction, such as PPI (Grover and Leskovec, 2016).

We use G=(V,E) to denote the GO graph, where *V* represents the set of GO terms and *E* all the undirected edges named ‘is_a’ and ‘part_of’ appearing between the terms. Node2vec seeks to optimize the objective Function 1, which maximizes the log-probability of observing the neighbourhood $N_{s}(u)$ of a node *u*, given its feature representation. The neighbourhood, *N_S_*, is defined by a biased random walk, which interpolates the BFS and DFS search strategies and the function *f* can be seen as a matrix of size |V|×d, where *d* is the chosen embedding size. Node2vec extends the objective function by making two standard assumptions: conditional independence and symmetry in feature space. The likelihood is factorized such that the likelihood of observing two different nodes in the neighbourhood is independent given the feature representation. Two nodes in the same neighbourhood also have a symmetry effect in the feature space. Node2vec is trained using stochastic gradient ascent with negative sampling to accommodate for large networks (Grover and Leskovec, 2016).

(1) $$\underset{f}{\max}\sum_{u\in V}\;\text{log}\;P(N_{S}(u)|f(u)).$$

To create continuous feature representations for proteins, we use $S=\{GO_{1},GO_{2},,,GO_{n}\}$ to denote the set of all GO terms that are annotated to a protein. Using the learned matrix defined by the function *f* from node2vec, we replace the GO IDs with the corresponding rows, such that the resulting set would be of size |S|×d.

We use the code of node2vec provided by the authors to train the model on the GO graph. The hyper-parameters used are summarized in Table 1.

**Table 1.** Node2vec hyper-parameters

Window/neighbourhood size	10
Walk length	80
Number of walks	10
Search bias P	1
Search bias Q	1
Iterations	10
Dimensions	64

### 2.2 The architecture of TransformerGO

**Fig. 1.**
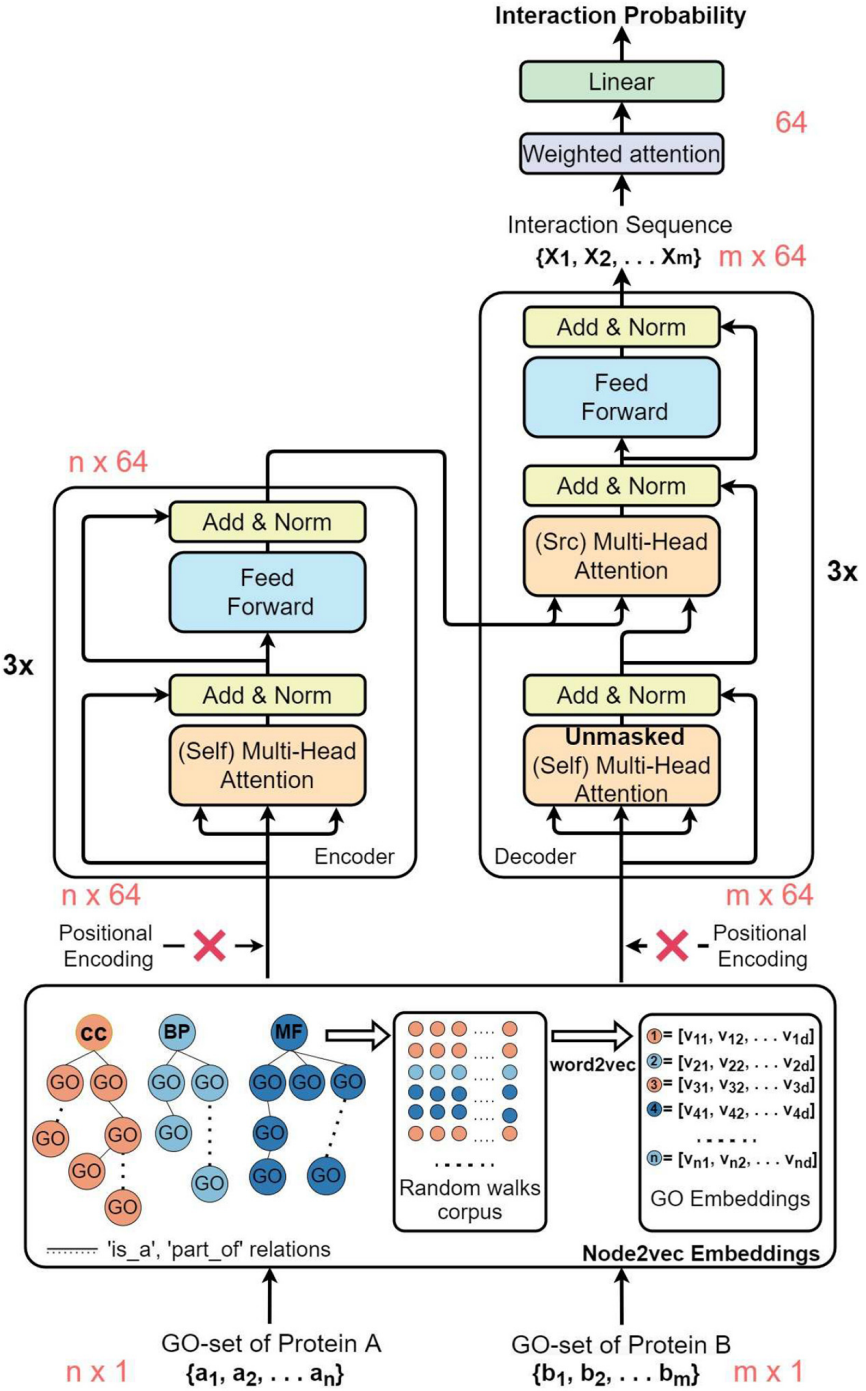
The architecture of our framework, with labels in red, highlighting the dimensionality of the data passing through the computational graph. The drawing is based on the original Transformer architecture. Positional encodings are not injected into the input GO-sets, and the mask for future positions is removed from the decoder. The lower part of the figure shows the generation of GO embeddings. The text corpus for training the word2vec model is created by performing biased random walks on the GO graph

We introduce a framework developed for GO-based PPI prediction, capable of analysing complex relations between sets of GO terms. The model proposed is based on the Transformer (Vaswani *et al.*, 2017), which uses an attention mechanism to solve seq2seq tasks, such as translation. In the recent years, multiple models have been published that use the Transformer architecture to achieve state-of-the-art results on natural language-processing tasks: the Vanilla Encoder is used to train deep bidirectional representations from unlabelled text by conditioning on both left and right context (Dai *et al.*, 2019; Devlin *et al.*, 2018; Liu *et al.*, 2019). While the Transformer architecture is designed for seq2seq tasks, with different modifications it can be used for biological prediction tasks. The model proposed by Lifan, TransformerCPI (Chen *et al.*, 2020), uses the Transformer Decoder to model the protein–compound interaction, while the Encoder is replaced by a set of convolution blocks. To the best of our knowledge, the Transformer model has not been used to predict PPIs at a functional level. Inspired by the ability of the model to capture deep connections between sequences, we developed TransformerGO to predict PPIs from sets of GO terms. An overview of the model proposed is in Figure 1, where we made modifications to the attention-heads and changed the final layers. 

Both the Encoder and Decoder receive as input an embedded set of GO terms, which are used to define the protein at a functional level. Through self-attention mechanisms, GO terms are weighted accordingly to the contribution they have as an interaction descriptor. Replacing the Masked Multi-Head Attention with a Multi-Head attention allows the model to attend to subsequent positions, a change that transfers the architecture from an autoregressive task to a classification task (Chen *et al.*, 2020). Given that the order of the GO terms should not be relevant in predicting interactions at the functional level, the positional encodings are not injected in our input, which enables annotated GO terms to be treated as a set.

The key component of the Transformer network is the Scaled-Dot-Product-Attention, which allows the model to focus on certain parts of the input. The attention function can be described as mapping a query and a set of key–value pairs to an output. In practice, the attention is computed on a set of queries using a matrix as follows:

(2) $$\mathrm{Attention}(Q,K,V)=\mathrm{soft}\;\max(\frac{QK^{T}}{\sqrt{d_{k}}})V,$$

where *Q*, *K* and *V* are matrices for the set of queries, keys and values and $\sqrt{d_{k}}$ is a scalar factor.

Multi-Head-Attention blocks allow the model to attend at different positions and subspaces by linearly projecting the input using learned weights before performing the attention function. This mechanism enables the Decoder to focus dynamically on certain parts of the Encoder’s output to learn semantic similarities between GO terms that could be important when predicting PPIs.

After extracting deep semantic similarities between GO terms, the output of the Transformer consists of an interaction sequence, which has the same shape as the input of the Decoder. We apply the same method used in TransformerCPI (Chen *et al.*, 2020) to transform this sequence into a final probability vector. Given the interaction sequence $\left\{X_{1},X_{2}\ldots X_{m}\right\}$, we modify the volume by using a weighted sum of the attention vectors. Therefore, the input to the final Linear layer is given by Equation (3), where $\mathrm{att}n_{i}=\frac{e^{||X_{i}|{|}_{2}^{2}}}{\sum_{j=1}^{M}e^{||X_{j}|{|}_{2}^{2}}},$
 

(3) $$\mathrm{input}=\sum_{i=1}^{M}{\text{attn}}_{i}X_{i}.$$

The entire architecture is trained to optimize the Binary Cross-Entropy Loss, given the binary nature of the PPI prediction:

(4) $$\mathrm{Loss}=-[y_{n}\;\text{log}\;x_{n}+(1-y_{n})\;\text{log}(1-x_{n})],$$

where *y_n_* is the label of the class and *x_n_* a function returning the predicted probability of class ‘1’.

TransformerGO was implemented in PyTorch (Paszke *et al.*, 2017) and trained using the Adam optimizer (Kingma and Ba, 2014). We reduced the size of the Transformer from six layers to three and the embedding size from 512 to 64, but we kept the number of heads to eight as this did not increase training time. The training hyper-parameters and the model settings are summarized in Table 2.

**Table 2.** TransformerGO hyper-parameters and settings

Embedding size	64
Number of layers	3
Number of heads	8
Feed forward dimension	64×4
Learning rate	1*e*−04
Batch size	32
Dropout	0.2

### 2.3 Datasets

#### 2.3.1 GO graph and GO annotation data

- *GO* *graph* (Ashburner *et al.*, 2000)—a filtered version of the ontology graph is downloaded, which guarantees that the generated graph is acyclic and there are no relationships that cross the three GO hierarchies. The file’s release date is September 19, 2018 to ensure a fair comparison with previous work.
- *GO annotation data—*we adopted the files provided by Jain’s work (Jain and Bader, 2010). Electronically inferred annotations that lack manual review are included and named IEA+.

#### 2.3.2 PPI datasets

**Jain’s datasets** (Jain and Bader, 2010)—contain positive and negative interactions for *Saccharomyces* *cerevisiae* and *Homo* *sapiens* organisms. A number of 4598 positive interactions and 2077 respectively, were retrieved using the Database of Interacting Proteins (Xenarios *et al.*, 2000). These were further split into three smaller datasets (BP, MF and CC) with both proteins annotated to terms (other than root) in their respective ontologies (Tables 3 and 4). For *S.cerevisiae*, an equal number of negative interactions are generated at random by selecting protein pairs from the GO annotation file that are not part of all known yeast PPIs appearing in the iRefWeb database (45 448 yeast PPIs) (Razick *et al.*, 2008). For *H.sapiens*, an equal number of negative interactions are generated at random from a pool of all possible interactions and then removing those which appear in the iRefWeb database (43 935 human PPIs).

**Table 3.** Distribution of positive interactions for *H.sapiens*

	IEA+			IEA−		
Ontology	**PPI**	**Self-PPI**	**Nodes**	**PPI**	**Self-PPI**	**Nodes**
CC	1387 + **44**	147	1115	1022 + **32**	125	832
BP	1391 + **44**	150	1119	1163 + **41**	135	920
MF	1397 + **44**	149	1119	1246 + **42**	140	978

*Note*: The bold PPIs represent redundant interactions, which appear in the dataset. The Nodes represent unique protein IDs that appear in the dataset. Self-PPI are interactions between the same protein.

**Table 4.** Distribution of positive interactions for *S.cerevisiae*

	IEA+			IEA−		
Ontology	**PPI**	**Self-PPI**	**Nodes**	**PPI**	**Self-PPI**	**Nodes**
CC	4469	194	2187	4425	190	2146
BP	4385	192	2123	4326	189	2076
MF	3858	177	1847	3583	173	1731

**STRING-DB datasets**: To analyse the performance of TransformerGO on larger datasets, we obtained two protein interaction networks for *S.cerevisiae* and *H.sapiens* from the STRING database (Szklarczyk *et al.*, 2016, downloaded on November 17, 2021), which account for 1 988 592 and 11 938 498 interactions. After filtering the interactions that have a score above 700 (high confidence), the *S.cerevisiae* dataset is composed of 120 386 interactions from 5966 proteins and *H.sapiens* is composed of 252 984 interactions from 16 814 proteins. We used gene annotation files for both organisms from the STRING website along with the GO graph. We filtered down annotations inferred electronically (IEA) and annotations where there is no biological data available. We generated an equal number of negative interactions by randomly choosing pairs of proteins from the positive dataset that do not appear in the STRING database (Table 5).

**Table 5.** Distribution of positive interactions for *S.cerevisiae* and *H.sapiens* in the STRING-DB dataset

Organism	PPI	Self-PPI	Nodes
*S.cerevisiae*	120 386	0	5966
*H.sapiens*	252 984	0	16 814

To allow for a fair comparison with previous methods, we also train and test the TransformerGO model on a benchmark containing 420 534 human interactions and 119 051 yeast interactions (Kulmanov *et al.*, 2021) retrieved from the STRING database. The difference between our proposed dataset and the benchmark is the use of up-to-date files and the method of generating negative interactions. We consider negative interactions, pairs of proteins for which there is no association in the STRING database, while the previous proposed benchmark considers interactions under the confidence score (700) to be negative. We also found the use of ‘mirror’ interactions where we consider both A→B and B→A interactions to be redundant for training, and we do not include them in our proposed datasets.

## 3 Results and discussion

### 3.1 The order of the input sequence

Zhang’s work (Zhang *et al.*, 2020) proposes a new method called *protein2vec* that differs from classic semantic similarity measures between GO terms. Here, a protein is characterized by a vector according to the annotated GO terms. A network embedding algorithm is applied to generate dense feature vectors for each GO term, then the resulting sequence of embeddings becomes input to a long short-term neural network. To model the interaction between two proteins, the outputs from the LSTM network are fed into a feed-forward neural network which outputs an interaction probability,

We argue that the annotated GO terms should not be modelled as a sequence due to the nature of the information they hold. GO terms are simple labels that should not act as words in a sentence. We re-implemented the model proposed by Zhang (Zhang *et al.*, 2020) and performed experiments by changing the order of the terms in the sequence. As shown in Table 6, there is a decrease in accuracy for all subsets, showing that the LSTM is modelling the interaction based on the order of the GO terms in the sequence. Therefore, some improvement in accuracy comes from the model’s bias over the ordering of the input.

**Table 6.** AUC values on *S.cerevisiae* from a 5-fold cross-validation experiment

	IEA+			IEA−		
	**CC**	**BP**	**MF**	**CC**	**BP**	**MF**
protein2vec	0.91	0.89	0.88	0.90	0.90	0.87
Sorted input*	0.921	0.915	0.917	0.899	0.912	0.916
Shuffled input*	0.905	0.897	0.895	0.888	0.894	0.892

*Note*: The values present in the table represent the mean AUC value. The standard deviation is <0.01 in all cases. The results highlighted with ‘*’ are from a re-implementation of *protein2vec*.

### 3.2 TransformerGO performance on Jain’s datasets

We choose two classic semantic similarity measures, TCSS and Resnik (Jain and Bader, 2010; Resnik, 1995), to compare performance on Jain’s datasets along with two more recent approaches, HVSM and protein2vec (Zhang *et al.*, 2018, 2020). We use the receiving operating characteristics (ROC) curve, which is a method used widely to measure the performance of binary classifiers. ROC is defined by plotting the true-positive rate (sensitivity) $\mathrm{TPR}=\frac{TP}{TP+FN}$ against the false-negative rate (1-specificity) $\mathrm{FPR}=\frac{FP}{FP+TN}$, where TP stands for the number of true-positives, FP for false positives and TN for true negatives. We report the area under the curve (AUC) as a measurement of performance (Fawcett, 2006). Note that semantic similarity measures do not require a training phase of the algorithm, therefore the validation results are reported on the entire dataset. To fairly compare our work with these measures, we perform a 5-fold cross-validation on *S.cerevisiae* and *H.sapiens* and report the average AUC values.

In Tables 7 and 8, the results of the proposed methods are shown, with the TransformerGO performance marked in bold. We can observe that classic semantic similarity measures along with the Hierarchical Vector Space Model perform relatively poor compared to machine learning models. TransformerGO improves the performance on average with 5% across all subsets, indicating that semantic similarities methods fail to capture the true meaning of the GO graph and use it to predict PPIs. While protein2vec uses a similar way of encoding GO terms as TransformerGO, the LSTM is designed to capture features from sequences where the order of the ‘words’ has a deep meaning. Furthermore, protein2vec only uses a feed-forward network to capture the semantic similarity features between two gene products. The decoder in our approach focuses its attention dynamically on the output of the encoder to capture interaction features of the two sets of GO terms.

**Table 7.** AUC values on *H.sapiens* from a 5-fold cross-validation experiment

	IEA+			IEA−		
	**CC**	**BP**	**MF**	**CC**	**BP**	**MF**
TCSS-max	0.82	0.92	0.85	0.80	0.89	0.80
Resnik-max	0.81	0.92	0.84	0.80	0.89	0.80
HVSM	0.84	0.93	0.88	—	—	—
Protein2vec	0.85	0.87	0.82	0.85	0.89	0.82
TransformerGO	**0.936**	**0.933**	**0.939**	**0.912**	**0.927**	**0.912**

*Note*: HVSM does not report the results on the datasets without electronically inferred annotations. The standard deviation of TransformerGO is <0.01 across all datasets.

**Table 8.** AUC values on *S.cerevisiae* from a 5-fold cross-validation experiment

	IEA+			IEA−		
	**CC**	**BP**	**MF**	**CC**	**BP**	**MF**
TCSS-max	0.83	0.89	0.75	0.83	0.89	0.73
Resnik-max	0.83	0.89	0.75	0.83	0.89	0.73
HVSM	0.83	0.90	0.74	—	—	—
Protein2vec	0.91	0.89	0.88	0.90	0.90	0.87
TransformerGO	**0.927**	**0.929**	**0.924**	**0.921**	**0.926**	**0.926**

### 3.3 TransformerGO performance on STRING-DB datasets

Taking into consideration that the Transformer network requires a large training corpus, and it is easy to overfit on small datasets (Qiu *et al.*, 2020), we trained our model on two considerable larger datasets retrieved from STRING database (Szklarczyk *et al.*, 2016). We randomly split the datasets into 80% training, 20% testing, and use 20% of the training dataset as a validation set to choose the best performing model. This would allow for a better analysis of the TransformerGO performance on an external test set and the effects of a larger training corpus.

We benchmark our model against recent work on generating feature vectors for GO terms and proteins. Onto2vec (Smaili *et al.*, 2018) is a method that learns representations for classes in an ontology and biological entities annotated with these classes. To generate feature vectors for GO terms and proteins, it trains a skip-gram model on the set of all axioms appearing in the GO. Opa2vec (Smaili *et al.*, 2019) extends this method by including meta-data from the ontology (natural language statements) into the training corpus and using transfer learning from biomedical literature. El Embeddings (Kulmanov *et al.*, 2019) embeds classes by minimizing a set of loss functions that preserve the axioms inside an ontology. In Table 9, we observe that the classic semantic similarity, such as Resnik outperforms some unsupervised methods. El Embeddings has good performance on the task of PPI prediction by exploiting more axioms from the ontology. Adding all the GO terms generated by node2vec together, combined with cosine similarity outperforms opa2vec. This suggests that most of the information contained in the GO can be captured by exploiting the graph neighbourhoods generated by the ‘is_a’ and ‘part_of’ relations. As it has been shown before (Smaili *et al.*, 2018, 2019), the main advantage of generating embeddings for GO terms is that it allows a downstream model to learn semantic similarity in a supervised way. TransformerGO improves AUC values on the *S.cerevisiae* and *H.Sapiens* STRING benchmark with up to 7% compared to the second-best method. Using a simple feed-forward neural network with three layers (200, 400 and 200 neurons) on top of the embeddings performs better than cosine similarity, but it fails to match the performance of our model. This again validates the importance of the Decoder in our network, which is able to capture deep semantic similarities between GO terms.

**Table 9.** AUC values on *S.cerevisiae* and *H.sapiens—*STRING benchmark

	*S.cerevisiae*	*H.sapiens*
Resnik	0.87	0.89
Onto2vec	0.80	0.77
Opa2vec	0.88	0.88
El Embeddings	0.93	0.90
Node2vec_COS	0.847	0.845
Node2vec_NN	0.952	0.958
TransformerGO	**0.961**	**0.974**

*Note*: The best AUC values are marked in bold. Node2vec_COS adds all the embeddings together and predicts PPI using cosine similarity. Node2vec_NN trains a simple feed-forward neural network on top of the embeddings.

Semantic similarity has been used to capture similarities between gene products from different perspectives. In the case of GO-based semantic similarity, the CC terms build up a context, which could be used to validate physical interactions and localization-dependant functions or processes. Furthermore, the BP aspect offers insight into indirect interactions of proteins involved in the same process network (Pesquita *et al.*, 2009). To investigate the (interaction) description power of each sub-ontology, we train and test TransformerGO on our dataset with features derived from CC, BP and MF sub-ontologies. Considering that there are no edges in the GO DAG between the sub-ontologies, there is no information leakage within the node2vec generated embeddings, allowing for a correct evaluation of the performance. In Table 10, we observe that the model which combines features from all the sub-ontologies performs consistently better than those trained on filtered datasets. This reflects the ability of the model to extract information from relations appearing between GO terms which belong to different sub-ontologies. Similar to previous work (Xu *et al.*, 2008; Zhang and Tang, 2016), we find that the performance for BP and CC is better than MF. However, proteins in the dataset have on average less MF annotations than BP and CC. This raises the question if annotation size has an effect on performance.

**Table 10.** AUC values on *S.cerevisiae* and *H.sapiens—*STRING dataset

*S.cerevisiae*				*H.sapiens*			
**CC**	**BP**	**MF**	**ALL**	**CC**	**BP**	**MF**	**ALL**
0.941	0.966	0.935	**0.973**	0.924	0.948	0.900	**0.958**

*Note*: Each column represents the AUC value of TransformerGO trained and evaluated on a filtered dataset with ALL or only GO terms from a specific sub-ontology. The bold values represent the top AUC results.

Protein interaction networks have been used to infer properties and functions of proteins through a ‘guilt by association’ principle, which states that proteins that are associated (interact) are more likely to have similar functions (Oliver, 2000). Recent studies (Gillis and Pavlidis, 2011, 2012) show that function can be extracted from interactions networks without using ‘guilt’ and only using the node degree as input. This proved to be successful because genes that have more interacting partners are more likely to have multiple functions (GO terms). To explore if TransformerGO is biased towards proteins with more annotations (multifunctional), we train and test the model using datasets filtered at different annotation sizes. We consider the annotation size to be the size of the GO-set containing all the GO terms annotated to both interacting proteins. In Table 11, we observe that in the most conservative case where interactions are defined by only up to six terms, there is a considerable drop in performance. This suggests that interactions between proteins with few known functions are difficult to predict, due to a decrease in chance of finding similar or semantic similar GO terms. For interactions with a higher number of GO terms associated, there is a clear improvement in AUC values. However, it is interesting to see that the performance drops when the model is trained and evaluated using interactions, which contain proteins with a high number of annotations (above 20 for *S.cerevisiae* and 30 for *H.sapiens*). This suggests that the model is not biased towards proteins with a high number of annotations, but capturing semantic similarity between proteins with a few annotations proves to be a difficult task.

**Table 11.** AUC values on *S.cerevisiae* and *H.sapiens—*STRING dataset

*S.cerevisiae*		*H.sapiens*	
**GO-set size**	**AUC**	**GO-set size**	**AUC**
**(0, 6)**	0.822	**(0, 6)**	0.711
**(0, 10)**	0.948	**(0, 10)**	0.840
**(10, 20)**	0.973	**(10, 30)**	0.953
**(20, ∞)**	0.963	**(30, ∞)**	0.951

*Note*: AUC values of TransformerGO trained and evaluated on a filtered dataset containing only interactions where the aggregated number of GO terms is within a specific range. The bold values represent the GO set size defining the interaction.

### 3.4 Model interpretation

One desirable outcome along a semantic similarity value between two proteins would be to determine what GO terms are more important and in what manner they relate to each other when predicting an interaction. For example, when modelling the interaction between two binding proteins, the CC should be more important than their ability to regulate other proteins (Smaili *et al.*, 2018). This is one of the reasons why negative interactions are usually generated to include proteins from different CCs or BPs (Bandyopadhyay and Mallick, 2017). Our understanding of the success behind the Transformer networks is limited, but recent work in the field of NLP brings light in the interpretation of attention (Kovaleva *et al.*, 2019; Rogers *et al.*, 2020). In the field of Bioinformatics, attention has been used to show how it captures the folding structure of proteins and target binding sites (Vig *et al.*, 2020). We follow this work to analyse the attention weights of TransformerGO and observe the patterns learned when modelling semantic similarity between sets of GO terms. We define an indicator function *f* that takes as input interacting proteins *A*, *B* and returns one if the pair of GO terms determined by the index pair (*i*, *j*) is present in the interaction, and zero otherwise. We compute the attention matrix *M* that aggregates the attention weights over all the GO term pairs in the ontology and all the interactions in dataset *X* as follows:

(5) $$M_{i,j}=\sum_{(A,B)\in X}f(A,B)\alpha_{i,j}(A,B)/\sum_{(A,B)\in X}f(A,B),$$

where $\alpha_{i,j}$ denotes the attention from GO term *i* to GO term *j* in the input interaction.

**Fig. 2.**
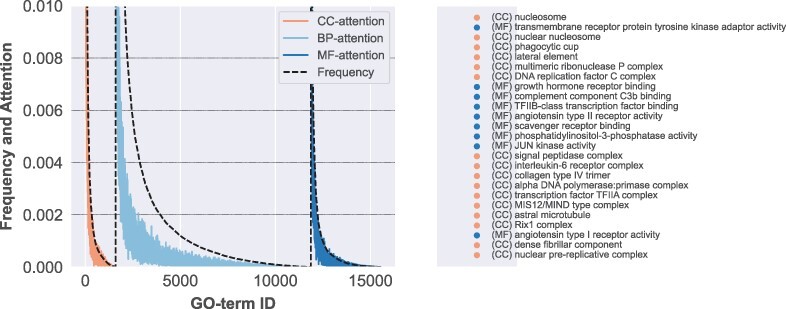
The frequency of each GO term that appears in the *H.sapiens* dataset (String database) along with the attention value, which is computed by summing over the columns of the matrix *M*. CC and MF terms have high attention values similar to the background frequency. On the other hand, BP terms have low attention values despite appearing frequently in the dataset. This demonstrates the ability of TransformerGO to ignore noise and focus on terms that are more important when predicting PPIs. On the right side of the figure, the top 25 terms where the attention value is higher than the background frequency are highlighted. Note that there are no BP terms that have this property

We use a model trained on the *H.Sapiens* STRING-DB benchmark dataset and analyse the attention using Equation (5). Compared to the training corpus used in NLP models, the dataset is relatively small, therefore, we only analyse the average attention over all layers and attention-heads. In Figure 2, we can observe that the attention of CC and MF terms is highly dependent on the background frequency in the dataset. This could suggest that the model is considering frequent terms a good indicator of interaction, due to an increase chance of finding semantic similarity between protein pairs. However, there is a considerable discrepancy in GO term frequency and attention values for BP terms: the Decoder allows the model to capture complex semantic relations between GO terms, disregarding information that comes in the form of noise, which does not contribute towards interaction prediction. On the right side of Figure 2, the top 25 terms that have attention values greater than the background frequency are shown. It is interesting that the model is paying more attention to CC terms, which define complex like structures. In other words, the interaction is easier to predict if we know that both proteins are part of the same complex. Similar for BP, a few terms which focus on the binding activity are being picked up. One important question that arrives is why does the model disregard most of the MF information, considering that training on this part of the ontology alone showed top performance. Our intuition suggests that TransformerGO finds it ‘easier’ to model terms, which define the interaction in broader terms than focussing on terms, which have a more granular view of a possible association between proteins.

**Fig. 3.**
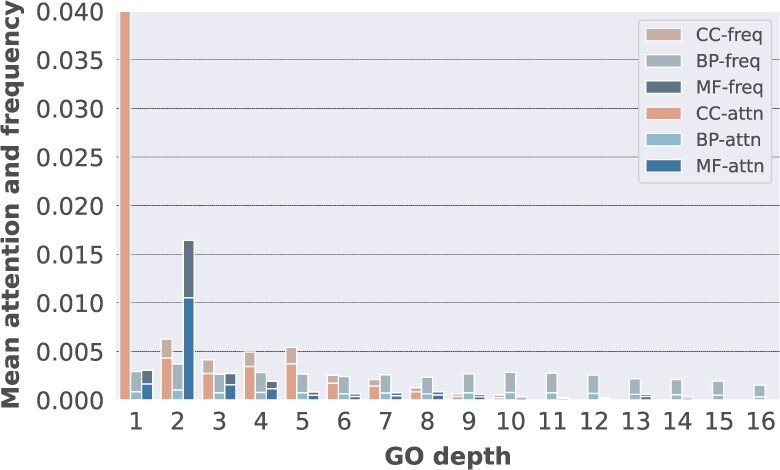
The mean attention of GO terms residing at different depths of the GO graph (the longest path to the root), along with the mean background frequency. There is a strong correlation between attention values and background frequency. CC terms, which are closer to the root are preferred, while terms from other ontologies have attention values distributed more equally

Previously, classic semantic similarity measure has been shown to be biased towards the depth of annotation classes (Kulmanov and Hoehndorf, 2017), with GO terms, which are more specific showing, on average, an increase in similarity score to other terms. To evaluate the effects of depth when it comes to modelling GO terms, we consider the depth as the longest path to the root of the ontology and analyse the attention accumulated per level. In Figure 3, we can see that the attention values are similar with the background frequency, suggesting that the model is not focussing on certain parts of the GO graph to extract information about protein interactions. Another interesting aspect is that TransformerGO ‘pays’ the same attention to all BP terms, regardless of the depth at which they appear in the GO graph. This shows again that BF terms are more granular and when used in isolation, they provide more information about protein interactions, therefore, achieving top performance. We also observe that in the case of CC and MF terms, there is high attention at the root of ontology, highlighting the fact that terms which define complexes act as ‘clique-like’ structures in contrast to other terms (Gillis and Pavlidis, 2012). In other words, GO terms, which have high learnability in the network data can act in ‘reverse’ and be used to predict interactions: CC terms, which define complexes and MF terms, which define binding processes.

**Fig. 4.**
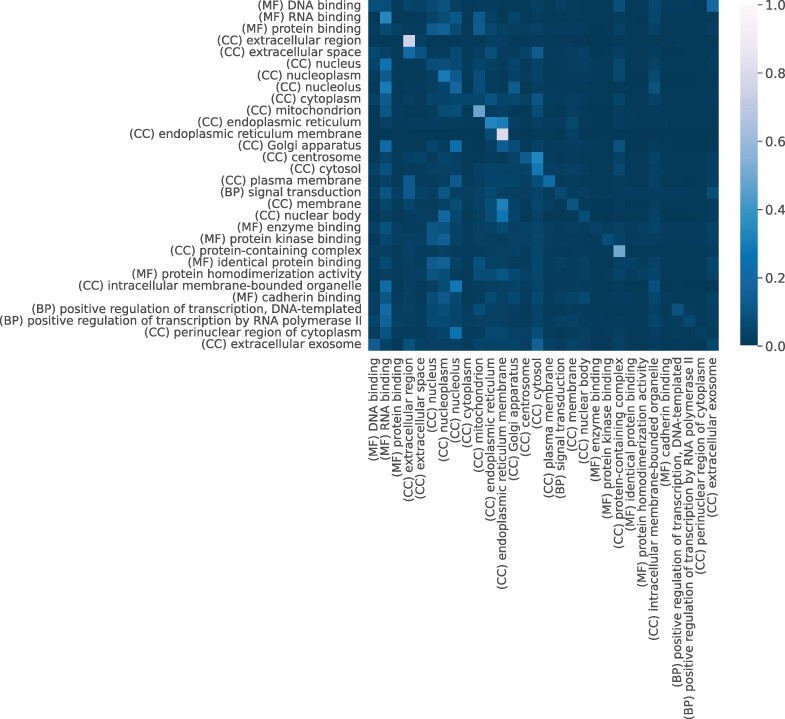
The attention values of the top 30 GO terms according to the information aggregated from the self-attention block of both the Encoder and Decoder when predicting human interactions experimental validated using ‘High Throughput’ methods. At this stage, ‘box like’ patterns appear on the diagonal, highlighting semantic similarity between terms that are one level apart

To further analyse what the model is paying attention to when predicting the interactions, we map the positive interactions from the *H.sapiens* dataset (String benchmark) to the BioGRID database (Oughtred *et al.*, 2019). This allows us to compare which GO terms are more significant in predicting the interaction depending on the type of experiment that determined it. A number of 23 923 interactions are labelled as ‘High Throughput’ and 11 729 as ‘Low Throughput’, while the rest of the interactions were not found in the BioGRID database. In Figure 4, we computed the attention heatmap using the self-attention weights when the model gets as input interactions labelled as ‘High Throughput’. We further filtered down the heatmap to contain only the top 30 GO terms that have high attention values. We can observe that a diagonal pattern appears similar to previous work of analysing BERT’s self-attention weights (Kovaleva *et al.*, 2019). This could suggest the model’s inability to capture semantic similarity at an early stage when the GO terms are analysed in isolation (the other protein in the interacting pair is not seen). However, there are similarities captured within the CC ontology between terms, which are one level apart: ‘Extracellular region’ → ‘Extracellular space’ and ‘Endoplasmic reticulum’ → ‘Endoplasmic reticulum membrane’. One common motif that appears across the heatmaps is the importance of GO terms that define binding functions, e.g. ‘DNA, RNA, protein, enzyme binding’. This could suggest that proteins, which have a binding behaviour are more likely to interact.

**Fig. 5.**
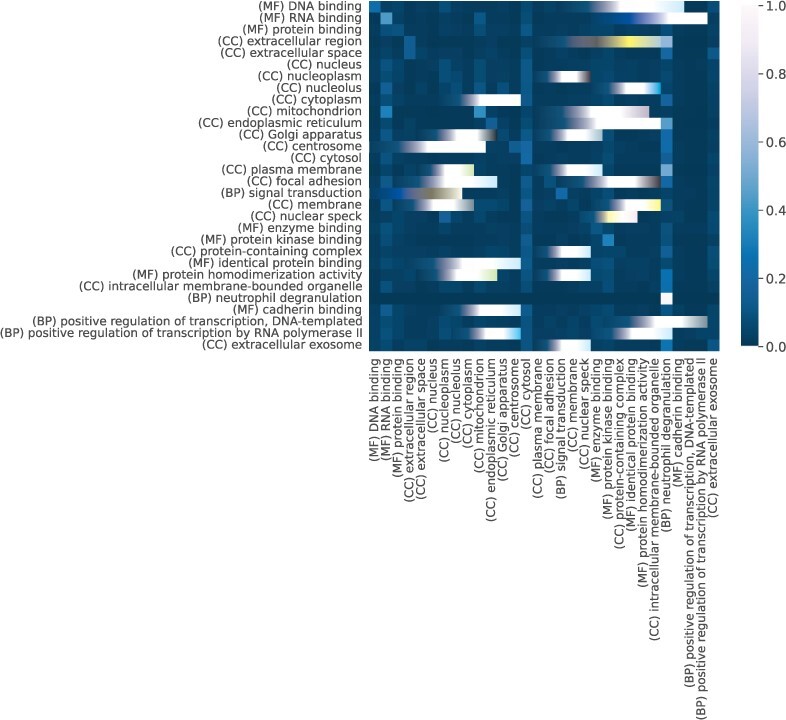
The attention values of the top 30 GO terms according to the information aggregated from the source-attention of the Decoder when predicting human interactions experimental validated using ‘High Throughput’ methods. The importance of binding terms is being picked up, along with the BP term ‘Neutrophil degranulation’

The heatmap in Figure 5 drawn from the source-attention weights highlights specific semantic relations between GO terms, which are part of different sub-ontologies. Such important terms appear as vertical line patterns, e.g. ‘(BP) Neutrophil degranulation’, ‘(CC) Cytosol’. Similar patterns appear in the attention heatmaps for yeast, e.g. ‘(CC) Nucleus’ and ‘(MF) structural constituent of ribosome’ (Supplementary Figs S1–S4).

**Fig. 6.**
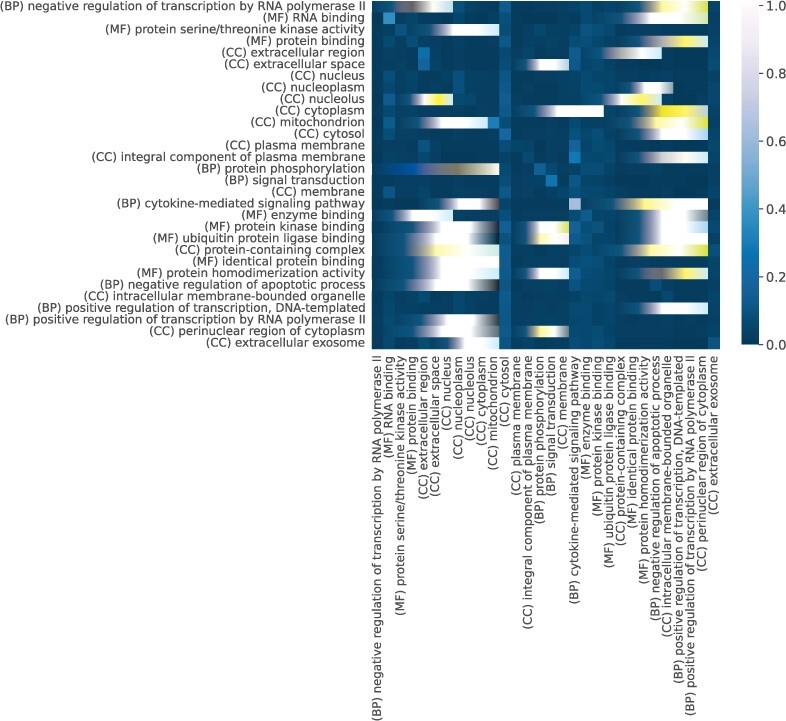
The attention values of the top 30 GO terms according to the information aggregated from the source-attention of the Decoder when predicting human interactions experimental validated using ‘Low Throughput’ methods. In this case, most of the attention is ‘paid’ to binding terms

The difference in important GO terms when predicting interactions determined by ‘Low Throughput’ methods compared to ‘High Throughput’ is minimal (see Fig. 6). In both cases, CC terms have high attention values even if the terms are close to the root of the ontology. While the background frequency of CC terms is higher than BP and MF terms, the model has the ability of disregarding unimportant terms. Therefore, the presence of large number of CC terms with high attention is not necessarily due to a background frequency. One explanation could be that the model views CC terms as valuable because proteins that are localized in the same CC are more likely to interact. This is similar to previous work (Shin *et al.*, 2009) that demonstrated that observed frequencies of co-location do not arise by chance.

## 4 Conclusions

Multiple methods that compute semantic similarity between GO terms have been proposed in the recent years, but the choice of appropriate use still depends on the application, as the performance can vary for different applications. Thus, they fail to answer which method is the most appropriate measure given the biological question (Mazandu *et al.*, 2017). Furthermore, classic similarity measures have been shown to be biased due to the number of annotations, difference in annotation size and depth of specificity of annotation classes when predicting PPIs (Kulmanov and Hoehndorf, 2017). We proposed TransformerGO, a method that uses recent advancement from Deep Learning to predict PPIs using network information extracted from the GO graph. One clear advantage of our model compared to semantic similarity measures is the ability to use generalized feature vectors of GO terms and then weight them accordingly in the training phase using an attention mechanism. This overcomes a limitation of manually created semantic similarity measures to judge how each relation between terms should contribute towards the end goal (Smaili *et al.*, 2018). TransformerGO improves performance compared to recent machine-learning approaches due to a careful design that captures the semantic similarity between GO sets. Onto2vec and Opa2vec (Smaili *et al.*, 2018, 2019) encode the GO terms similarly as TransformerGO, but the prediction of the interaction is modelled by a simple cosine similarity or a shallow fully connected neural network. While protein2vec (Shin *et al.*, 2009) uses an LSTM to model the protein representation, the input is considered a sequence of terms and the interaction is still predicted by a fully connected layer.

A new trend is to allow these methods to take into consideration modern high-throughput technologies from various datasets (Mazandu *et al.*, 2017). TransformerGO takes as input feature vectors of GO terms and can be trained to solve other biological questions, such as predicting the type of interactions between protein pairs.

Transformers are neural networks that use attention to accelerate training (Vaswani *et al.*, 2017) and are the main component of state-of-the-art NLP architectures, such as BERT (Devlin *et al.*, 2018). Interpreting attention is an active and well-known area of research (Jain and Wallace, 2019; Wiegreffe and Pinter, 2019), but the application to biological sequences is still lagging behind. We propose a visualization of the attention-heads that extends previous work to the field of semantic similarity-based prediction of protein interactions. Unlike classic semantic similarity measures, the source-attention offers valuable insights that explain the similarity between GO terms. We find that CC terms are an important indicator of interacting proteins and that there are cases where semantic similarity is being picked up across different ontologies.

While we demonstrated TransformerGO’s performance on the task of PPI prediction, semantic similarity is still far from reaching the status of other similarity measures between gene products, such as the sequence-based ones (Pesquita *et al.*, 2009).

We expect future research on attention based models to offer more comprehensive analysis of protein to protein interactions, thorough model interpretation of the semantic similarity at a more granular level.

## Funding

This work was supported by a studentship from the Engineering and Physical Sciences Research Council (EPSRC) via the University of Southampton. M.N.’s contribution is partially funded by EPSRC grant ‘Artificial and Augmented Intelligence for Automated Scientific Discovery’ [EP/S000356/1].

*Conflict of Interest*: none declared. 

## Supplementary Material

btac104_supplementary_data
