# Supplementary material for: TransformerGO: predicting protein–protein interactions by modelling the attention between sets of gene ontology terms
Source: Bioinformatics. 2022 Feb 17;38(8):2269–77. doi: 10.1093/bioinformatics/btac104 (PMC9363134; doi:10.1093/bioinformatics/btac104)
Supplement: btac104_supplementary_data [file btac104_supplementary_data.pdf]

Bioinformatics
doi.10.1093/bioinformatics/xxxxxx
Advance Access Publication Date: Day Month Year
Manuscript Category

OXFORD

Supplementary material

TransformerGO: Predicting protein-protein interactions by modelling the attention between sets of gene ontology terms

Ioan Ieremie [1,*], Rob M. Ewing [2], and Mahesan Niranjan [1]

[1]Vision, Learning & Control Group, University of Southampton, Southampton, SO17 1BJ, UK and
[2]Biological Sciences, University of Southampton, Southampton, SO17 1BJ, UK.

Abstract

Motivation: Protein-protein interactions (PPIs) play a key role in diverse biological processes but only a small subset of the interactions have been experimentally identified. Additionally, high-throughput experimental techniques that detect PPIs are known to suffer various limitations such as exaggerated false positives and negatives rates. The semantic similarity derived from the Gene Ontology (GO) annotation is regarded as one of the most powerful indicators for protein interactions. However, while computational approaches for prediction of PPIs have gained popularity in recent years, most methods fail to capture the specificity of GO terms.
Results: We propose TransformerGO, a model that is capable of capturing the semantic similarity between gene ontology sets dynamically using an attention mechanism. We generate dense graph embeddings for GO terms using an algorithmic framework for learning continuous representations of nodes in networks called node2vec. TransformerGO learns deep semantic relations between annotated terms and can distinguish between negative and positive interactions with high accuracy. TransformerGO outperforms classic semantic similarity measures on gold standard PPI datasets and state-of-the-art machine learning-based approaches on large datasets from S. cerevisiae and H. sapiens. We show how the neural attention mechanism embedded in the transformer architecture detects relevant functional terms when predicting interactions.
Availability and implementation: https://github.com/Ieremie
Contact: ii1g17@soton.ac.uk

© The Author 2015. Published by Oxford University Press. All rights reserved. For permissions, please e-mail: journals.permissions@oup.com

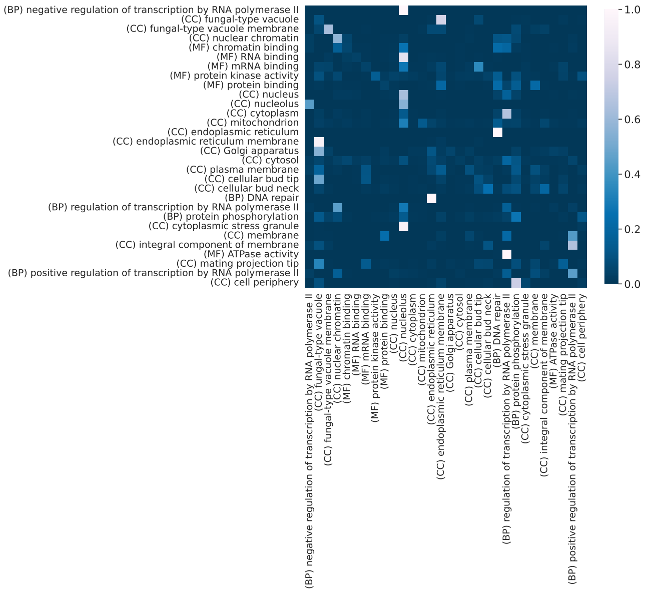

Fig. 1. The attention values of the top 30 GO terms according to the information aggregated from the self-attention block of both the Encoder and Decoder when predicting yeast interactions experimental validated using 'High Throughput' methods.

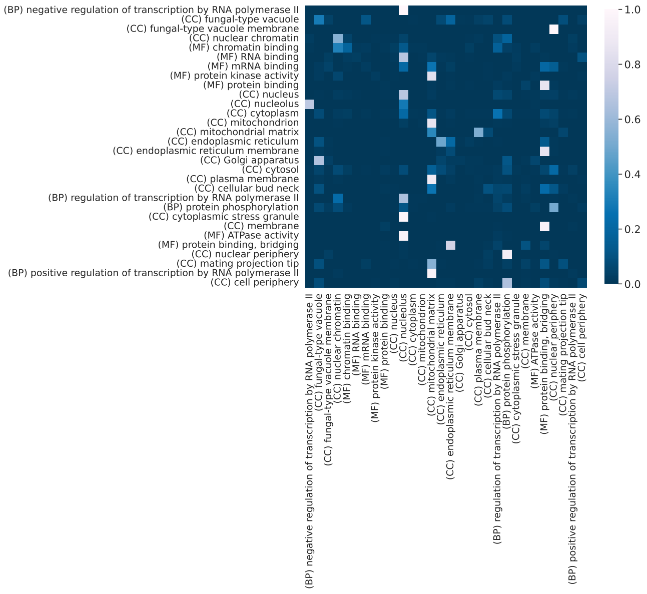

Fig. 3. The attention values of the top 30 GO terms according to the information aggregated from the self-attention block of both the Encoder and Decoder when predicting yeast interactions experimental validated using 'Low Throughput' methods.

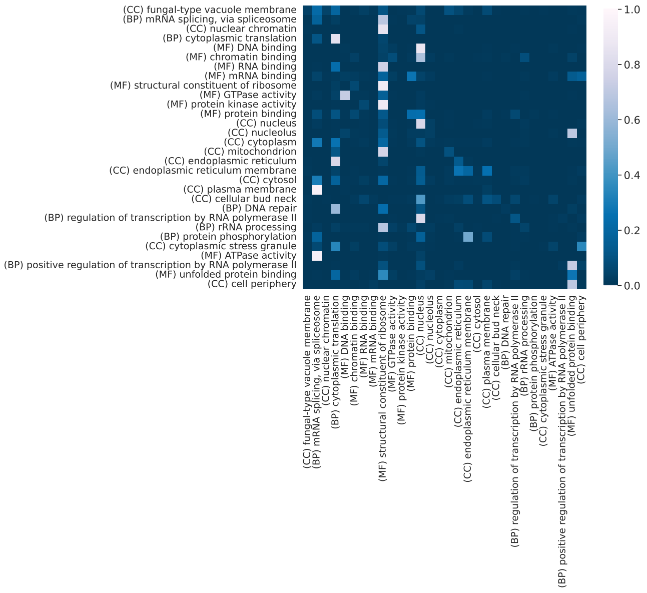

Fig. 2. The attention values of the top 30 GO terms according to the information aggregated from the source-attention of the Decoder when predicting yeast interactions experimental validated using 'High Throughput' methods.

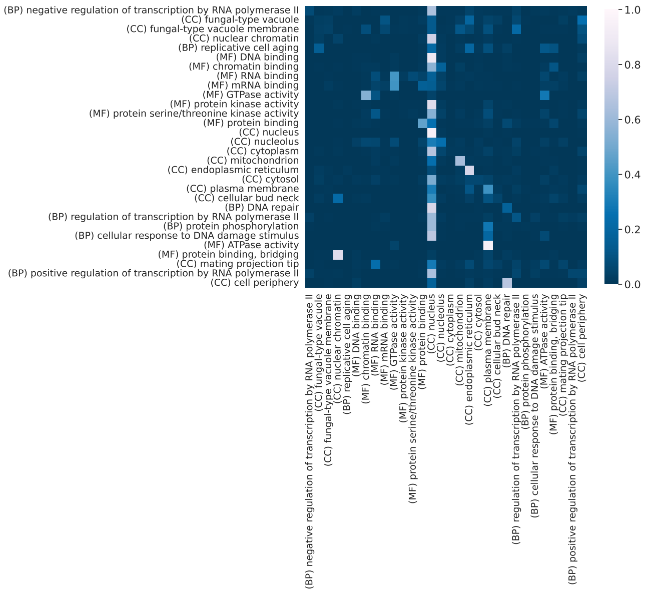

Fig. 4. The attention values of the top 30 GO terms according to the information aggregated from the source-attention of the Decoder when predicting yeast interactions experimental validated using 'Low Throughput' methods.
